# Association between different dimensions of anger and symptoms of post-traumatic stress disorder in at-risk cardiovascular patients during the COVID-19 pandemic

**DOI:** 10.3389/fpsyt.2023.1228192

**Published:** 2023-09-27

**Authors:** Mihailo Nesic, Julia Vogel, Jan Philipp Krüger, Werner Wenzel, Ali Sahebi, Tienush Rassaf, Johannes Siebermair, Ulrich Wesemann

**Affiliations:** ^1^Department of Psychiatry, Psychotherapy and Psychotraumatology, Bundeswehr Hospital Berlin, Berlin, Germany; ^2^Department of Cardiology and Vascular Medicine, West German Heart and Vascular Center Essen, University Duisburg-Essen, Duisberg, Germany; ^3^Department of Microbiology, Bundeswehr Hospital Berlin, Berlin, Germany; ^4^Psychosocial Injuries Research Center, Ilam University of Medical Sciences, Ilam, Iran; ^5^Non-Communicable Diseases Research Center, Ilam University of Medical Sciences, Ilam, Iran; ^6^Department of Cardiology, Krankenhaus Göttlicher Heiland GmbH, Vienna, Austria

**Keywords:** PTSD, anger, COVID-19, cardiology, hospital, pandemics, cardiovascular diseases, heart diseases

## Abstract

**Introduction:**

The common connecting factor between PTSD and cardiovascular diseases lies in the disruption of the stress processing system. The COVID-19 pandemic has led to an increase in stress levels worldwide. Due to the life-threatening situation of affected risk patients, this also led to the accumulation of post-traumatic stress symptoms (PTSS). The influence of anger on cardiovascular diseases has hardly been investigated so far. The focus of this study is on anger regulation in cardiovascular risk patients. The COVID-19 pandemic is considered as an additional stressor in this study, but not as a separate entity. The hypothesis is that individuals with inward anger are more prone to post-traumatic stress disorder (PTSD).

**Methods:**

As part of the routine examination, all patients who were hospitalized between January 1st, 2021 and May 31st, 2022 with high-risk cardiovascular diseases were included. A total of *N* = 153 (84.1%) subjects participated in the study. On admission, anger (STAXI-2) and PTSD (PCL-5) were assessed using questionnaires. The relationship between different domains of anger and PTSS was examined.

**Results:**

Inwardly directed anger was more pronounced in this population than in a standard sample (+1 SD) and had a significant impact on the presence of PTSD (B = −0.72, *p* < 0.001). Additionally, correlations were found between inward-directed anger and PTSD, as well as all other anger expressions studied and the PTSD total score.

**Discussion:**

It can be assumed that anger and its regulation are relevant factors for both cardiac diseases and PTSD. The study results can be used for prevention, rehabilitation and therapeutic measures. However, the impact of inner anger on PTSD is theoretical and based on statistical testing. A confirmatory longitudinal study is needed to substantiate these results.

## 1. Introduction

Epidemiological data indicates that the common mechanisms between PTSD and cardiac disease are routed through the stress dissipation system (1). These links between trauma, PTSD and cardiac diseases have been confirmed by the extensive Adverse Childhood Experiences (ACE) study. The more such experiences (traumas) were or are present, the greater the risk for cardiological diseases (2, 3).

PTSD, depression and anxiety increase the risk of heart failure (4, 5). Post-Traumatic Stress Disorder has a 47% increased risk of developing heart failure within 7 years (6). The common underlying mechanisms are disorders and dysfunctions in the autonomic nervous system (7) with hyperactivity in the sympathetic system and concomitant hypoactivity in the parasympathetic system (8). The other neuropsychological mechanisms run via the brain-heart axis. These pathways run through the frontal, limbic brain areas and the autonomic nervous system (9). PTSD patients show higher catecholamine concentrations and an increased heart rate compared to people who are not affected by the condition (10). That increased heart rate is an important factor in the recurrence of acute coronary events (11). The studies mentioned here, as well as other scientific work, clearly show that there are several common causes as well as mutual interactions between cardiac diseases and PTSD.

The other key association between PTSD and cardiac diseases lies in the psychological factors (12). Edmondson et al. studied a large cohort of patients with acute coronary syndrome and PTSD for the risk of recurrence of coronary symptoms. Among other things, they found that medication non-adherence with beta-blockers can lead to a recurrence of myocardial infarction. This is due to the imbalance of the autonomic nervous system in the first three months after the acute coronary event (12).

HPA axis dysregulations with disturbances in cortisol secretion are another significant mechanism (13). These dysregulations contribute to the development of cardiac diseases (1). In addition, PTSD is associated with endothelial dysfunction (14).

Disease-promoting factors in heart disease include management of negative emotions (15), propensity to anger and hostility (16), inhibitions to express negative emotions (17), and a low socioeconomic status or social adversity (18). On the other hand, these psychological factors may pose a susceptibility to the development of PTSD in cardiac patients.

In earlier studies, an influence of a strong tendency to anger on PTSD and differences in the expression of anger between patients with and without PTSD could be determined (19).

The main symptom groups of PTSD consist of intrusion, traumatic avoidance, hyperarousal, and persistent negative alterations in cognition and mood that began or worsened after the trauma (20, 21). PTSD-symptoms are strongly associated with various negative emotions, especially anger (22). Anger can be described as a negative inner state (23). This condition is associated with deviations in perception and cognitive judgment. Resulting cognitive distortions include increased blaming of others, feelings of increasing injustice, or a tendency to avoid (23). The increase in anger due to traumatic experiences has also been demonstrated in several studies (24–26). In addition, it has been found that different domains of anger, such as B. the trait anger, are pre-traumatic risk factors for PTSD (27).

A meta-analysis shows that low social support and loneliness are associated with a higher risk of coronary artery disease (28). Loneliness also increased during the pandemic (29). According to several studies, the COVID-19 pandemic has a negative impact on various psychological issues, such as stress, anxiety and anger (30). COVID-19 represents an increased psychological load. Research sees the pandemic as a cultural trauma - due to the loss of trust in general as well as in social institutions and the loss of one’s existential safety (31). These processes also increase the risk of developing various diseases, including PTSD. According to the Norwegian study by Bonsaksen et al. (32), PTSD has been found in 12.5% of men and 19.5% of women in the general population. Galea et al. also look at the pandemic in relation to the increase in PTSD numbers (33). Based on this, it can be concluded that the corona pandemic is a risk factor for PTSD.

The aim of this study is to examine hospitalized patients with cardiovascular risk diseases during the COVID-19 pandemic for PTSD and various forms of anger. A COVID-19 infection is not the subject of the investigation, but the pandemic is regarded as an additional stressor. Of interest is the impact of expressions of anger, particularly internal anger, on susceptibility to PTSD. In addition, other forms of anger expression will be distinguished between patients with and without PTSD and the relationship between anger expressions in relation to the presence of specific symptom clusters of PTSD will be examined. Our assumption was that patients with internal expressions of anger were more likely to suffer from PTSD.

## 2. Materials and methods

The data collection took place between January 1, 2021 and May 26, 2022 in the West German Heart and Vascular Center of the University Hospital Essen for Cardiology and Angiology. All patients who were admitted to Essen University Hospital due to cardiovascular conditions were included in the study. All patients filled out several questionnaires as part of routine clinical diagnostics. From 182 possible participants aged 18 and over, data were obtained from *N* = 153 risk patients (inclusion rate 84.1%). The main reasons for non-participation were immobility and lack of concentration. The main reasons for admission were: coronary artery disease (*n* = 33; 21.6%), heart failure (*n* = 32; 20.9%), atrial fibrillation/flutter (*n* = 34; 22.2%), pulmonary disease (*n* = 17; 11.1%), diabetes (*n* = 12; 7.8%), peripheral artery disease (*n* = 12; 7.8%%) and others (n = 13; 8.5%). The ages ranged from 19 to 92 years (mean 62 years, median 63 years). On admission, *n* = 5 (3.3%) patients had a COVID-19 infection, *n* = 6 (3.9%) patients came for the first time for a cardiovascular condition. All patients signed provided informed consent. The study was approved by the local ethical committee (number: 22-10982-BO).

### 2.1. Psychometric test procedures

STAXI-2: With the State–Trait Anger Expression Inventory-2 (STAXI-2), different aspects of anger can be recorded. In certain situations, anger can be defined as state anger or trait anger (anger as a personality trait) in its various forms of expression (directed inwards or outwards). The same applies to anger control (34). The following dimensions are recorded in the questionnaire: inwardly directed anger, open expression of anger, degree of anger, situational anger, anger reaction to criticism or evaluation by others, verbal and physical anger impulse form, anger as temperament or tendency to anger and rage and various forms of anger control, such as anger control through internal calming or external prevention.

The retest reliability scales are divided into: state anger between RTT = 0.14 and 0.29, trait anger between RTT = 0.67 and 0.78, and the anger expression and anger control scale between RTT = 0.63 and 0.81. The internal consistency scale is between α = 0.79 and 0.91 on the anger trait scale and between α = 0.80 and 0.90 on the anger expression and anger control scales. Extensive knowledge is available on the validity of the STAXI-2. Various studies prove the factorial validity of the method (35).

PCL-5: The Posttraumatic Stress Disorder Checklist PCL-5 in the German version serves as a survey method for recording post-traumatic stress symptoms and provisional PTSD diagnoses according to DSM-5 and ICD-11 criteria (36). The PCL-5 is a test procedure with 20 items, which are divided into PTSD symptoms on a Likert scale from 0 “not at all” to 4 “extremely.” The temporal criterion of a PTSD diagnosis includes the last month. The test was developed based on the DSM-5 criteria. The investigations showed a high internal consistency (α = 0.94) as well as high discriminability and convergence. In addition, there was good retest reliability of 0.56 < *R* < 0.82 (37). For the German version, the findings were substantiated by a corresponding validation on a civilian and military sample (38). A total score of 33 points was used to indicate the presence of PTSD (36). In the test description, the information “Think of your worst event” is given. The critical incident (Criterion A) was assessed by the question “What incident do you think of, your heart disease [x] or another [x] [….].

### 2.2. Statistical testing procedures

Our sample’s anger scales were compared to a standard sample published by the test authors (35). A deviation of ± one standard deviation (SD) of the norm sample was defined as a significant deviation. The influence of inwardly directed anger on the presence of PTSD was tested using linear regression analysis (PTSD sum score) and binary logistic regression analysis (PTSD yes/no). The predictor variable “inner anger” was previously tested for a violation of homoscedasticity. The dependent variable was the PCL total score or the presence of PTSD (PTSD yes/no). For further differentiation, an ANCOVA was performed to test the influence of “inner anger” on the PCL total score including the covariates gender, age and critical incident (dichotomized in heart condition or other). Afterwards, Pearson’s correlation coefficients between inward-directed anger and the severity of PTSD symptom clusters were calculated. In addition, the association between the dimensions of anger and overall PTSD symptoms was determined using Pearson correlations. Due to the small number of patients with COVID-19 and patients with initial admission, no separate calculation was carried out for these subgroups (Table 1).

**Table 1:** *t*-test for independent samples to test for gender differences in age and PTSD symptoms.

	Sex	*N*	Mean	*SD*	*T*-value	df	*p*-value
Age	Female	77	62.2	15.86	0.209	149	0.835
	Male	74	61.6	18.67			
PCL-5 (Scale)	Female	78	29.4	12.69	−0.420	151	0.675
	Male	75	30.2	12.05			

SD, standard deviation; df, degree of freedom; *p*-value: significance level 2-tailed; PCL-5 (Scale): Total PTSD symptom score as measured by the Post-Traumatic Stress Disorder Checklist. Differences in df are due to missing data.

## 3. Results

All anger scales in our sample were within 1 SD of the norm sample, but inwardly directed anger was 1 SD higher. According to PCL-5, PTSD was suspected in *n* = 48 (31.4%) patients. When describing the critical incident, n = 108 (70.6%) indicated their heart disease. The logistic regression analysis revealed a significant positive influence of inwardly directed anger on PTSD symptoms with *R*^2^ = 0.22, *F*(1, 138) = 39.1, *p* < 0.001. Using binary logistic regression analysis, a significant positive influence of inwardly directed anger on the presence of PTSD was demonstrated with a non-standardized beta weight *B* = −0.72, standard error = 0.18, Wald = 15.8, *p* < 0.001. The odds ratio was 1.14 (95% confidence interval, 1.06–1.22). This means that the likelihood of suffering from post-traumatic stress disorder increases by 14% with each higher point on the Inward Anger Scale.

An ANCOVA testing the impact of “internal anger” on the PCL total score including the covariates gender, age, and critical incident confirmed this impact with *R*^2^ = 0.40, as shown in Table 2.

**Table 2:** ANCOVA to test the influence of inwardly directed anger on PTBS symptoms including gender, age and critical incident (heart condition yes/no) as covariates.

Predictors	*df*	*F*	Sig.	*R* ^2^	Ƞ^2^
Gender	1	0.37	0.545		0.003
Age	1	0.02	0.877		<0.001
Incident	1	0.20	0.653		0.002
inwardly directed anger	24	2.89	<0.001		0.386
Total	138			0.396	

Df, degrees of freedom; *F*, *F*-value, Sig., significance; *R*^2^, coefficient of determination; ƞ^2^, partial eta square.

There was also a positive correlation between PTSS total score and all anger modalities studied. A detailed representation can be found in Table 3.

**Table 3:** Pearson’s correlation between the sum score PCL-5 and the anger dimensions of the STAXI-2.

		Feeling angry	Feel like expressing anger verbally	Feel like expressing anger physically	State anger	Angry temperament	Angry reaction	Trait anger	Anger expression out	Anger expression-in	Anger control-out	Anger control-in	Anger expression index
PCL-5 Scale	Pears. Corr.	0.426	0.324	0.259	0.372	0.293	0.347	0.387	0.198	0.470	0.219	0.274	0.274
	Sig. (2-tailed)	< 0.001	< 0.001	0.002	< 0.001	< 0.001	< 0.001	< 0.001	0.019	< 0.001	0.009	0.001	0.001
	*N*	138	139	138	139	142	140	142	141	140	141	141	142

In addition, there was a significant association between inward-directed anger and each PTSD cluster. The detailed results are given in Table 4.

**Table 4:** Pearson’s correlation between inward-directed anger and the PTSD symptom clusters.

		Cluster B	Cluster C	Cluster D	Cluster E
Inward-directed anger	Pearson-Korrelation	0.371	0.374	0.395	0.414
	Sig. (2-tailed)	<0.001	<0.001	<0.001	<0.001
	N	133	129	139	135

Cluster B: “Intrusion, negative memories”; Cluster C: “Avoidance of external and internal triggers”; Cluster D: “Negative beliefs about self and others, negative emotions”; Cluster E: “Hyperarousal”.

## 4. Discussion

In the present study, the focus was placed on the correlations between different anger domains and PTSS. Regarding the different expressions of anger, the highest correlation was found between PTSS and inward-directed anger. These are the subjects who experience strong anger but cannot show it openly. Accordingly, the results also confirmed our assumption that the more the anger is directed inwards, the more likely the presence of PTSD is. This result remained stable when gender, age, and critical incident were accounted for using ANCOVA.

Dealing with anger and its effects on health has long been known from psychocardiology. A study from the mid-90th showed that increased verbal expression of anger was associated with a better prognosis for coronary artery disease (39). Hostility and internal anger are associated with the severity of angina pectoris and the number of myocardial infarctions (40).

A cohort study of over 47,000 Swedish adults showed that anger can contribute to the development of cardiovascular disease and increased mortality (41). Our findings of increased inward-directed anger in patients with cardiovascular conditions are consistent with previous results. In a prospective study, Denolett and colleagues found an influence of suppressed anger on cardiac death or myocardial infarction (42).

To our knowledge, this is the first study to describe the effects of internal anger and PTSD in patients with cardiovascular conditions. However, another study found a significant link between PTSD and anger. Difficulty controlling anger has been found to be associated with PTSD. However, no correlation was found with respect to the other anger domains such as trait anger, status anger, or anger expression (43). In contrast, in the present study, all areas of anger showed a significant correlation with PTSD. The differences could possibly be that our patient’s underlying heart conditions have already led to higher levels of anger. In addition, stresses caused by the pandemic could also have contributed to this development.

The lowest correlation found in the present study was between PTSD and overt expressions of anger, which manifested as increased aggressive behavior, criticism, or insults toward other people. This result makes it clear that better expression of anger can contribute to a reduction in PTSD. In contrast, inner anger increases the likelihood of PTSD. The relevance of anger regulation was also demonstrated in a 2021 cohort study. A correlation was found between feelings of anger and its aggressive expression with an increased risk of death from cardiovascular and oncological diseases. The study also suggests that not only experiencing negative emotions but also managing them or managing anger may be important for some health outcomes (44). Anger has also been identified as a trigger of acute coronary syndrome (45) as well as a risk factor for cardiovascular disease (46).

A 2013 study of rugby players showed a link between anger and activation of the molecular immune mechanism. This activation led to increased expression of the genes encoding the cytokines (IL-1ß). The authors recommended considering and further researching cytokines as a biological marker of anger (47). Another study with military personnel found an influence of hostility (48) and PTSD on the expression of TNF-α receptors (49).

The further results of the present study show an association between PTSD both with situational anger (*r* = 0.37, *p* < 0.001) and with a stable tendency toward anger (*r* = 0.38, *p* < 0.001). As a result, prolonged anger leads to chronic activation of the autonomic nervous system. This finding is also supported by a study in which functional cMRI showed increased amygdala activity during a fear response in both men and women with PTSD compared to controls (50). Anxiety is one of the most well-studied emotions associated with PTSD. In the course of this, the question arises as to the relevance of the other emotions in PTSD, since these have not yet been sufficiently researched. This is particularly true of anger as a symptom of PTSD, which may need to be viewed as a distinct entity (51).

In the present study, significant correlations were found between inward-directed anger and individual PTSD clusters (clusters B, C, D, and E), with the highest value being found in cluster E, hyperarousal. One explanation could be that inner anger can express itself as hyperarousal.

A multiple regression analysis using disaster relief workers after the September 11, 2001, terrorist attack in the United States showed a significant association between anger and PTSD (52). Similar results were also found after the terrorist attack in Berlin in 2016 (53). This significant connection could also be demonstrated in another study with Vietnam veterans (54).

Hardly any new studies on different anger domains in PTSD have been published in recent years. A 2016 publication showed that anger as a personality trait is more pronounced in people with a mental illness than in the general population (55). This shows the need to further investigate anger in the context of PTSD, cardiovascular and oncological diseases.

## 5. Conclusion

Anger is an important emotion in PTSD. As this study shows, turning inward anger has an impact on the presence of PTSD. Additionally, correlations were found between all anger modalities and overall PTSD symptoms. It follows that a more detailed understanding of this emotion is relevant for research and practical psychotherapeutic application. Assessing anger level and anger regulation can be a useful predictor for better risk assessment of developing PTSD immediately after trauma.

On the other hand, anger can also be seen as a pre-traumatic risk factor. In occupations with an increased risk of PTSD, such as others, such as military, fire, police, and medical personnel, may consider regular voluntary anger regulation assessments to prevent the development of PTSD. Likewise, the expression of anger in high-risk patients could be improved preventively through low-threshold programs. Similar interventions have shown positive effects in reducing hostility associated with coronary artery disease. Psychological interventions have reduced diastolic blood pressure (56). Psychotherapy can also be used effectively in the treatment of psychological symptoms associated with coronary artery disease (57).

In addition, the regulation of anger is a relevant therapy focus for symptom reduction in existing PTSD. Future research is warranted in the precise recording of this evolutionarily relevant emotion. This both in its origin and in terms of its importance in PTSD treatment. The outcome could be a more targeted therapeutic application in clinical practice as previously suggested (58). However, initial attempts to address this could only achieve slight to moderate effects (59). On the other hand, a benefit of anger interventions was found in subjects with anger problems. You can therefore also benefit from such interventions before starting trauma-specific therapy (60) because anger has been shown to be a relevant factor in PTSD therapy (61).

A deeper understanding of the mechanisms of anger and its psychological and somatic effects on health could contribute to an improvement in the overall quality of life in the future. Resource-enhancing programs have also proven helpful (62). Dealing with aggression may also improve other conditions as it is also associated with a higher prevalence of workplace violence against healthcare workers (63).

Increased psychocardiological cooperation appears to make sense, as many overlaps have so far only been considered from “one” perspective.

### 5.1. Limitations

The study data were collected by means of questionnaires and not by means of interviews. However, interviews remain the gold standard for diagnosis. The prevalence tends to be overestimated in questionnaires. This is attributed to the specificity of the questionnaires (64). The results should be verified in a replication study. An additional limitation is the study design. Since the participants could only be examined at one point in time, the direct influence of inwardly directed anger on the presence of PTSD can only be statistically proven on the basis of hypotheses. Here, too, a replication study would be necessary that tests the presence of anger for the development of PTSD. The same applies to the influence of anger on the development of coronary heart disease. Of the patients admitted for the first time, five out of six reported their heart disease as a critical event, two of whom received a provisional diagnosis of PTSD. For this small subgroup (n = 2) it is possible that the diagnosis of PTSD is misleading as they may not meet the time criterion despite the past 30 days listed on the test.

## Data availability statement

The raw data supporting the conclusions of this article will be made available by the authors upon reasonable request.

## Ethics statement

The study was approved by the local ethical committee (University of Essen, Germany, number: 22-10982-BO). The patients/participants provided their written informed consent to participate in this study.

## Author contributions

UW and JS designed and guided the study. MN performed the literature screening, data analysis and interpretation of the results, and prepared the manuscript. JV collected the data and interpreted the results. JK, WW, AS, and TR revised the manuscript. All authors discussed and agreed on the final submitted version of the manuscript.
